# Valuing injection frequency and other attributes of type 2 diabetes treatments in Australia: a discrete choice experiment

**DOI:** 10.1186/s12913-018-3484-0

**Published:** 2018-08-30

**Authors:** Simon Fifer, John Rose, Kim K. Hamrosi, Dan Swain

**Affiliations:** 1 Community and Patient Preference Research (CaPPRe), Level 5, 478 George St, Sydney, NSW 2000 Australia; 2 Business Intelligence & Data Analytics (BIDA) Research Centre, University of Technology of Sydney, Ultimo, NSW 2007 Australia; 3 Swain Health Economics, 15 Tidal Cr, Moonee Beach, Coffs Harbour, NSW 2450 Australia; 4 Community and Patient Preference Research Pty Ltd, PO Box 1156, Darlinghurst, NSW 1300 Australia

**Keywords:** Discrete choice experiment, Diabetes, Willingness to pay, Patient preference, Consumer surplus, Reimbursement, Health technology assessment

## Abstract

**Background:**

Multiple pharmacotherapy options are available to control blood glucose in Type 2 Diabetes Mellitus (T2DM). Patients and prescribers may have different preferences for T2DM treatment attributes, such as mode and frequency of administration, based on their experiences and beliefs which may impact adherence. As adherence is a pivotal issue in diabetes therapy, it is important to understand what patients value and how they trade-off the risks and benefits of new treatments. This study aims to investigate the key drivers of choice for T2DM treatments, with a focus on injection frequency, and explore patients’ associated willingness-to-pay.

**Methods:**

A discrete choice experiment (DCE) was used to present patients with a series of trade-offs between different treatment options, injectable and oral medicines that were made up of 10 differing levels of attributes (frequency and mode of administration, weight change, needle type, storage, nausea, injection site reactions, hypoglycaemic events, instructions with food and cost). A sample of 171 Australian consenting adult T2DM patients, of which 58 were receiving twice-daily injections of exenatide and 113 were on oral glucose-lowering treatments, completed the national online survey. An error components model was used to estimate the relative priority and key drivers of choice patients place on different attributes and to estimate their willingness to pay for new treatments.

**Results:**

Injection frequency, weight change, and nausea were shown to be important attributes for patients receiving injections. Within this cohort, a once-weekly injection generated an additional benefit over a twice-daily injection, equivalent to a weighted total willingness to pay of AUD$22.35 per month.

**Conclusions:**

Based on the patient preferences, the importance of frequency of administration and other non-health benefits can be valued. Understanding patient preferences has an important role in health technology assessment, as the identification of the value as well as the importance weighting for each treatment attribute may assist with funding decisions beyond clinical trial outcomes.

## Background

Type 2 Diabetes Mellitus (T2DM) is a progressive condition in which a person’s body becomes resistant to the normal effects of insulin and/or slowly loses the ability to produce enough insulin in the pancreas causing elevations in blood glucose [1]. Worldwide it is estimated that 422 million people are living with diabetes or approximately 8.5% of the adult population [2]. In Australia, approximately 849,000 (4.7%) of adults aged 18 years are estimated to have T2DM, a higher rate among men than women, estimated at 5.4% versus 4.2% in 2016 [3]. The disease is associated with risk factors such as blood pressure, high cholesterol and coagulation. More seriously, it is also associated with reduced life expectancy and quality of life, driven by significant morbidity due to microvascular and macrovascular complications [4, 5].

Although there is no cure for T2DM, treatments typically use multiple interventions. Common treatment approaches focus on lifestyle modification followed by various pharmacotherapy options selected on the basis of individual clinical circumstances [6]. Optimising blood glucose control is necessary to improve both short- and long-term health outcomes in patients with T2DM. Those patients with uncontrolled or poor glycaemic control on one or more oral glucose-lowering agents may be treated with injectable treatments such as insulin and/or glucagon-like peptide-1 agonists (GLP-1 agonists). In Australia, prescribers have multiple treatment options to control their patient’s blood glucose, including oral and injectable treatments. These treatments play a vital role in the management of T2DM, controlling symptoms, preventing complications and improving health outcomes [5].

In respect to treatment, adherence remains a pivotal issue, with rates of adherence ranging from 36 to 93% among patients [7]. The determinants of non-adherence fall into the following categories: patient factors, treatment regimen, disease factors, prescriber-level factors (including patient-physician relationship), and the clinical setting. All evidence suggests the determinants of non-adherence in T2DM patients are consistent with other chronic conditions, although some may argue that insulin use is associated with unique barriers given its requirement for subcutaneous injection [8, 9]. As such, understanding patients’ preferences for the attributes of treatments, particularly mode and frequency of administration, and their relative importance to patients may result in tailored therapeutic decisions by prescribers that improve patient adherence, provide greater patient satisfaction and ultimately better health outcomes [10, 11].

The substantial body of research on patients’ treatment preferences into T2DM adult patients utilises a variety of methodologies such as discrete choice experiments (DCEs) [12], qualitative research [13], contingent valuation [14], revealed preference [15], time trade-off and other types of ranking and rating techniques [16]. Discrete choice experiments are a sophisticated way to assess the desirability and value of different attributes that do not exist in real markets as well as new alternatives. Previous patient preference literature has predominantly focused on attributes including efficacy, dosing schedule, glucose control, body weight change, and adverse events such as nausea and hypoglycemia [17, 18]. Although a few studies have focused on differing modes of administration, few have considered a cost attribute to determine patients’ willingness to pay for specific treatment attributes [12, 17, 19].

Thus, the aim of this study was to investigate the key drivers of choice for T2DM treatments, with a focus on valuing injection frequency and mode of administration, and to explore patient willingness to pay (WTP) for the different attributes of these treatments. Identifying patients’ preferences for these attributes may provide a basis for valuing the non-health benefits, aid in clinical decision-making as well as funding and reimbursement decisions.

## Methods

The study focused on the development of a choice modelling approach designed to gain an understanding of the treatment preferences of patients currently taking either twice-daily injectable exenatide (Byetta®) or oral glucose-lowering medicines in comparison to hypothetical new treatments. The study did not receive formal ethics approval as the online survey research did not impose, or was considered of minimal risk to participants, and only enrolled competent adults.

### Discrete choice experiment

The central feature of the study was the discrete choice experiment. DCEs were first developed in the late 1920s allowing for comparisons of two alternatives and later extended through theory and modelling from the1960s [20–22]. DCEs are now used in many fields to understand and model the trade-offs and preferences revealed by the choices that people make. Examples of the use of DCE methods include determining the rate of uptake for vaccinations prior to the vaccine going to market, community preferences for alternative policies on reducing smoking, and the valuation of alternative drug treatments.

### Survey instrument

The research involved the development of a survey with DCE. The attributes and levels of the DCE (Table 1) were based on previous qualitative research provided, an interrogation of patient preference literature, evidence from clinical trials of exenatide and consultation with external advisers (health economics and medical experts) and clinicians.

**Table 1:** An Overview of the Key Attributes

Attributes	Attribute levels
Frequency	Twice a day
	Once a day
	Once a week
	Once a month
Weight change (% body weight)	Lose 10%
	Lose 5%
	None
	Gain 5%
Needle you use to inject	Shorter / thinner (between a faint pain sensation and no pain sensation)
	Longer / thicker (a very weak pain sensation)
Storage	Keep in the fridge until first use
	No refrigeration required
Nausea	None
	Mild
	Moderate
Mode of administration (device)	Multi use pen (no mixing required)
	Multi use pen (some mixing required)
	Single use pen (no mixing required)
	Single use pen (some mixing required)
	Vial and syringe (no mixing required)
	Vial and syringe (some mixing required)
Injection site reactions / nodules	Yes
	No
Hypoglycaemic events per month	0
	1
	2
	> 2
Cost per script (month)	plus 60% (100% for oral)
	plus 45% (75% for oral)
	plus 30% (50% for oral)
	plus 15% (25% for oral)
	0
	minus 15%
	minus 30%
	minus 45%
Instructions with eating	Taken / used within a certain time of eating
	Taken / used anytime (unrestricted)

**Fig. 1:**
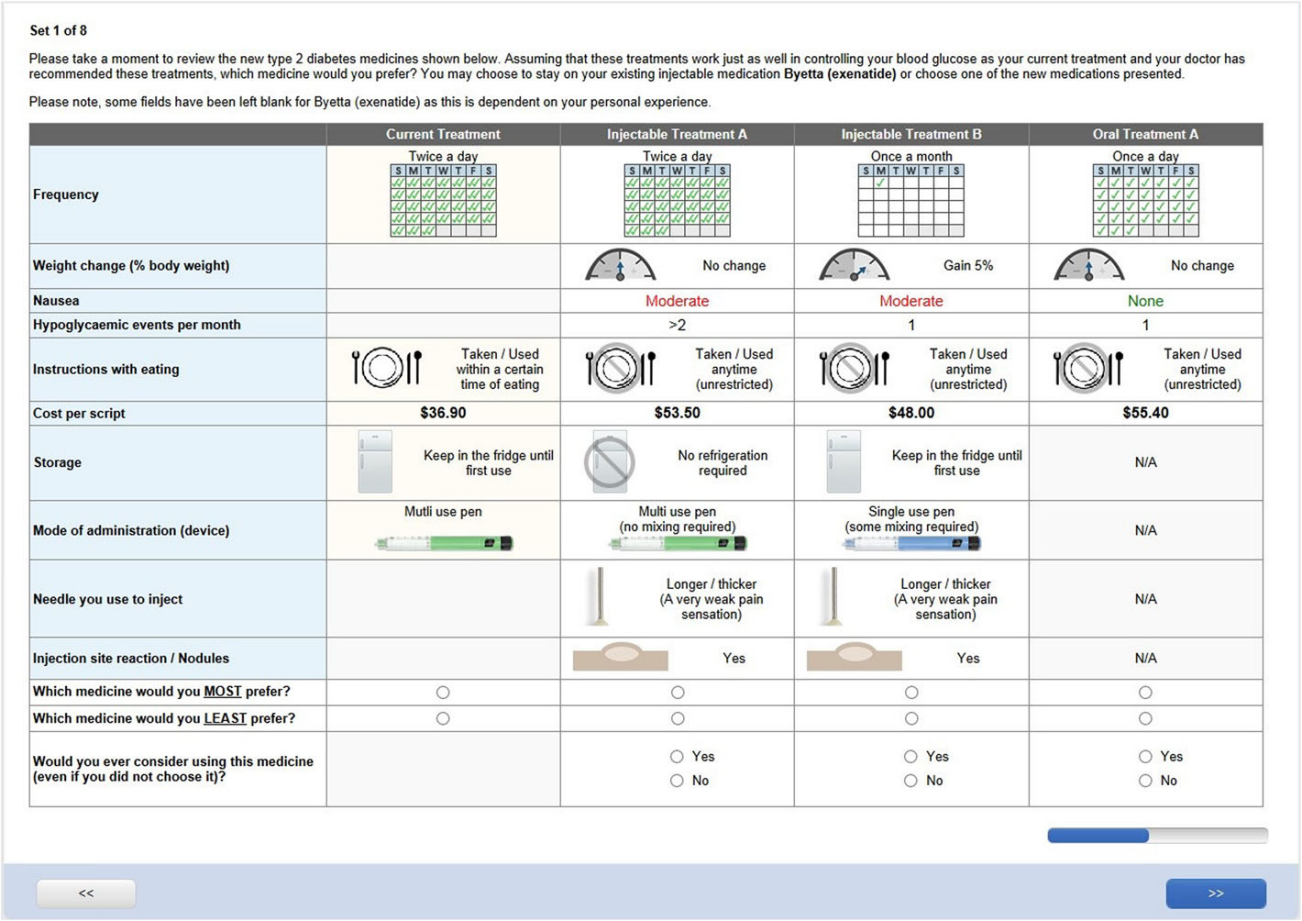
Example choice task for participant using exenatide

**Fig. 2:**
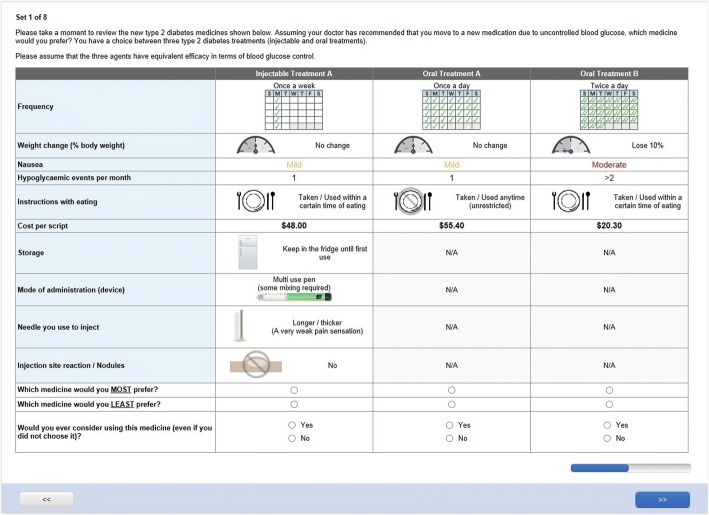
Example choice task for participant taking oral glucose-lowering medicine

The survey instrument consisted of seven sections (Table 1 broadly covering treatment experiences, satisfaction, quality of life, work productivity and preferences for treatment. The DCE component for the injectable cohort offered three hypothetical alternatives of treatment (either injectable or oral), plus a status quo alternative (exenatide) (Fig. 1). The oral cohort experiment was structured the same way except without the status quo alternative (Fig. 2). Respondents in each cohort were shown multiple hypothetical scenarios requiring them to select their preferred option from a set of competing alternatives (choice tasks). The features of the alternatives were then systematically varied, allowing for a determination of how each of the features impacts upon the preferences of each cohort. Participants each completed eight choice tasks which were randomised across participants.

A pilot study with 10 patients from each cohort were conducted prior to launching the main field phase to determine whether any changes to the content or design of the DCE were required. No changes were made to the overall survey and DCE as participants reported the attributes were relevant and the survey easy to understand.

### Study sample

For this study, the sample was stratified by current treatment to form two patient cohorts: 1) current patients using injectable exenatide treatment and 2) current patients on oral glucose-lowering medicines. A target sample size of a minimum 50 patients in each cohort was required to allow for sub-group analyses. To be eligible for the study, patients met the following criteria: 18 years of age or older, diagnosed with T2DM and on medicine for their T2DM, either injectable exenatide or oral glucose-lowering medicines.

Participants were mainly recruited through online panel providers as consumer panels are a cost effective and efficient way to recruit respondents nationally for online projects. Participants completed the survey in November 2014 and received reward points from the online panel provider. Supplementary recruitment was undertaken through advertising in local newspapers in capital cities to target eligible patients with T2DM. Interested patients were provided with contact details to enroll in the study.

### Willingness to pay

The literature identifies two primary types of WTP measures that can be derived from models of discrete choice; marginal WTP and total WTP. In this study, we concentrated on total WTP (also known as consumer surplus) which is the monetary representation of utility, or satisfaction-based economic value, accruing to the community. Accordingly, total WTP captures the additional benefits an alternative brings to a market and is often interpreted as the total value of the alternative. For example, the value of non-health outcomes (e.g. frequency or mode of administration) in supplementary cost-benefit analysis presented to regulatory / healthcare governing bodies [23, 24]. Thus, the change in total willingness to pay (or consumer surplus) is a comparison of two scenarios (e.g. market or policy changes etc.), drawn from the DCE [25]. This approach employs the definition offered by Train (2009 p. 56), shown in eq. (1).

1 $$ \varDelta E(CS)=\frac{1}{-{\beta}_{Cost}}\left[\mathit{\ln}\left(\sum \limits_{j=1}^{J^{New}}\ {e}^{V^{New}}\right)-\mathit{\ln}\left(\sum \limits_{j=1}^{J^{Current}}{e}^{V^{Current}}\right)\right] $$

Where ${e}^{V^{New}}$ is the exponential of the part-worth utility (hereinafter referred to as utility) for the new alternative, ${e}^{V^{Current}}$ is the exponential of the utility for the current alternative and *β*_*Cost*_ is the parameter for cost.

Applied to patients with T2DM in this study, the total WTP calculated represents the additional value this group of patients gains through improvements in new treatments compared to current treatments.

### Statistical analysis

Descriptive statistics were performed for each of the patient cohorts using SPSS. The combinations of levels for each attribute in the DCE were designed using the latest experimental techniques developed by Rose and Bliemer [26]. A Bayesian D-efficient design with basic priors to account for the direction of each parameter was used to structure the DCE and implemented in NGene version 1.1.2 (ChoiceMetrics Pty Ltd), a software tool used to generate stated choice experimental design. Bayesian efficient designs allow researchers to estimate suitable models with smaller sample sizes. All models were estimated using the specialised choice analysis software Nlogit 5.0 (Econometric Software Inc., NY, USA).

Whilst the simplest discrete choice model is called the Multinomial Logit model (MNL), this has certain restrictive assumptions related to error terms, independence of observed choices and homogeneity of preferences [22]. More advanced models such as the Error Components (EC) model, used to estimate the model in this study, allow for the easing of some of these assumptions [27]. As such, the EC model is a more flexible and superior model, allowing for correlation in the errors of the alternatives (assumption that errors are independently and identically distributed) and repeated choice observations (assumption that all observations are treated as independent even if they are from the same respondent).

Alternative specific parameters were estimated for injectable and oral treatments in this model. Further, different cost parameters were permitted to account for variation in price ranges based on concession status. Separate error components were estimated for the injectable alternatives [28]. Variables that were not significant were removed for the final model.

## Results

A total of 171 adult patients with T2DM completed an online survey in November 2014 assessing their preference for T2DM treatments and their willingness to pay for the different features of these treatments. Of those that completed the online survey, 153 were recruited through online panels and 18 through advertising. The study participants included 58 patients receiving twice-daily injections (injectable cohort) and 113 patients on oral treatments (oral medicine cohort). The sample size obtained was sufficient to estimate the models applied in this study given the attributes and levels in the experimental design.

Descriptive statistics of the patient characteristics for each of the two patient cohorts are presented in Table 2. The sample characteristics were similar across both cohorts with the exception of the oral medicine cohort being slightly older and more often entitled to a concessional rate for their medicines. The overall sample consisted of a greater percentage of males than females and the majority (78%) of participants were over 50 years of age. Almost half of all participants had a household income of $72,999 and under. Given the age of the study sample, this may suggest participants were retired and/or on an old-age pension.

**Table 2:** Background Socio-demographics

	Overall (*n* = 171) N,%	Injectable patients (*n* = 58) N,%	Oral patients (*n* = 113) N,%
Gender			
Male	104 (60.8)	37 (63.8)	67 (59.3)
Female	67 (39.2)	21 (36.2)	46 (40.7)
Age			
18–29 years	4 (2.3)	3 (5.2)	1 (0.9)
30–39 years	10 (5.8)	8 (13.8)	2 (1.8)
40–49 years	24 (14.0)	8 (13.8)	16 (14.2)
50–59 years	48 (28.1)	19 (32.8)	29 (25.7)
60–69 years	64 (37.4)	17 (29.3)	47 (41.6)
70 years and over	21 (12.3)	3 (5.2)	18 (15.9)
Household income			
$Nil-$33,799 ($0–$649 a week)	35 (20.5)	7 (12.1)	28 (24.8)
$33,800–$72,999 ($650–$1399 a week)	47 (27.5)	23 (39.7)	24 (21.2)
$72,800–$129,999 ($1400–$2499 a week)	45 (26.3)	15 (25.9)	30 (26.5)
$130,000–$181,999 ($2500–$3499 a week)	13 (7.6)	6 (10.3)	7 (6.2)
$182,000 or more ($3500 or more a week)	5 (2.9)	2 (3.4)	3 (2.7)
Prefer not to answer	26 (15.2)	5 (8.6)	21 (18.6)
Household composition			
Couple family with no children	60 (35.1)	15 (25.9)	45 (39.8)
Couple family with children	44 (25.7)	19 (32.8)	25 (22.1)
One parent family	6 (3.5)	2 (3.4)	4 (3.5)
Other family	5 (2.9)	1 (1.7)	4 (3.5)
Single person household	40 (23.4)	17 (29.3)	23 (20.4)
Group household (i.e., shared)	16 (9.4)	4 (6.9)	12 (10.6)
State			
ACT	4 (2.3)	2 (3.4)	2 (1.8)
NSW	77 (45.0)	22 (37.9)	55 (48.7)
VIC	41 (24.0)	19 (32.8)	22 (19.5)
QLD	18 (10.5)	4 (6.9)	14 (12.4)
SA	17 (9.9)	7 (12.1)	10 (8.8)
WA	10 (5.8)	1 (1.7)	9 (8.0)
TAS	3 (1.8)	3 (5.2)	0 (0.0)
NT	1 (0.6)	0 (0.0)	1 (0.9)
Patient type			
General Patient	89 (52.0)	36 (62.1)	53 (46.9)
Concessional Patient	82 (48.0)	22 (37.9)	60 (53.1)

### EC model outputs

The model results are shown in Table 3 below. Due to the small sample sizes, the exenatide and oral medicines patient samples were pooled and differences between these patient segments were tested using interaction terms. Alternative specific parameter estimates were estimated for both injection and oral treatments. Insignificant parameters (*p* > 0.05) were removed from the final DCE model. The model fit results illustrate that the model provided a superior fit to a constant only model. Main effects are estimated for both injectable and oral alternatives.

**Table 3:** Model Output for T2DM

Main effects	Injectable treatment		Oral treatment	
	*Parameter*	*t-ratio*	*Parameter*	*t-ratio*
Frequency				
Once a month	1.449	9.39	1.421	8.77
Once a week	1.192	8.05	1.374	7.5
Once a day	N/S	N/S	0.597	3.09
Twice a day	reference	reference	reference	reference
Weight change (% body weight)				
Lose 10%	0.698	8.62	2.257	1051
Lose 5%	1.217	8.61	1.737	7.48
None	1.450	3.73	0.582	2.11
Gain 5%	reference	reference	reference	reference
Needle you use to inject				
Longer / thicker (very weak pain sensation)	N/S	N/S	N/A	N/A
Shorter / thinner (faint pain sensation)	reference	reference	reference	reference
Storage				
Keep in the fridge until first use	N/S	N/S	N/S	N/S
No refrigeration required	reference	reference	reference	reference
Nausea				
None	0.641	3.74	0.610	3.93
Mild	0.368	2.14	0.273	1.65
Moderate	reference	reference	reference	reference
Mode of administration (device)				
Multi use pen	N/S	N/S	N/A	N/A
Single use pen	N/S	N/S	N/A	N/A
Single use vial and syringe	reference	reference	reference	reference
Injection site reactions / nodules				
Yes	N/S	N/S	N/A	N/A
No	reference	reference	reference	reference
Hypoglycaemic events per month				
0	0.521	3.11	N/S	N/S
1	N/S	N/S	N/S	N/S
2	N/S	N/S	N/S	N/S
> 2	reference	reference	reference	reference
Instructions with eating				
Taken / used within a certain time of eating	N/S	N/S	N/S	N/S
Taken / used anytime (unrestricted)	reference	reference	reference	reference
Cost				
Cost (General)	−0.015	2.27	−0.029	−7.25
Cost				
Cost (Concession)	N/S	N/S	−0.267	−4.6
Interaction effects (oral sample)				
Weight change * (oral)				
Lose 10%	N/S	N/S		
Lose 5%	1.239	4.24		
None	0.848	3.03		
Gain 5%	reference	reference		
Cost (concession) * (oral)				
Cost * sample			0.207	3.48
Sample (oral) constant			1.216	2.93
Constant				
Oral constant (concession)			1.816	3.52
Error components				
Injectable alternative (General)	1.446	6.36		
Injectable alternative (Concession)	−2.545	6.56		

Log likelihood (c): - 1438; Log likelihood (β): − 1031; Rho squared: 0.28; Number of respondents: 171; Number of choice observations: 1368

Abbreviations: *N/S* (Not Significant), *N/A* (Not Applicable)

The model results suggest that patients are mainly concerned with frequency, weight changes and nausea. Interaction effects (i.e. test for differences in preferences between injectable and oral patient sample) are also included. Oral patients are more concerned about weight changes and intuitively more likely to choose oral treatments.

### Relative attribute importance

In standard choice models, the parameters cannot typically be directly compared because the variables they are related to are presented on different scales, meaning the parameters also reflect different scales (e.g., the range of the cost attribute compared to other attributes). In this case, the attributes were multi-level categorical coded, so the magnitude of the parameter reflects the influence on utility. A Decision Support System (DSS) was used to further evaluate the importance of bundles of attributes by changing treatment combinations and observing the change in willingness to pay.

The top three attributes of importance were the same for patients in the injectable and oral medicine cohorts. These were in rank order: (i) weight change, (ii) frequency of administration and (iii) nausea. For patients using injectable exenatide, hypoglycaemic events per month ranked fourth, however this was non-significant in the oral medicine cohort. All other attributes were not significant.

### Willingness to pay

In this study, total WTP was calculated by comparing the utility difference (as per eq. 1) in two scenarios based on injection frequency differences. In these calculations the current scenario treatment was a twice-daily injection and the new scenario treatment was a once-weekly injection, which resulted in a weighted total WTP of $22.35 per month, holding all other attributes constant. The $22.35 total WTP can be interpreted as a monetary measure of the additional benefit or utility that patients gain from moving from a twice-a-day injection (current exenatide treatment) to a once-weekly injection (new treatment).

### Decision support system

A DSS was developed to support the visualisation of the model results. This enables the user (e.g., pharmaceutical company or clinician) to perform ‘what if’ scenarios based on hypothesised changes to the treatments, such as to frequency or side effects. In this research, willingness to pay or consumer surplus was a main outcome of interest in support of a reimbursement application. In Australia, the Pharmaceutical Benefits Advisory Committee guidelines allow for supplementary analysis where a medicine may have a direct patient benefit that is not a health outcome – as in this case, providing a more convenient form of administration to the patient.

The DSS was designed to reflect two dimensions of the injectable market - willingness to pay calculations for patients on injectable (current treatment) and for those moving to an alternative injectable treatment (new treatment). The results derived from this type of model may assist industry to better understand what patients value or prefer when developing new treatments as well as guide clinicians when making decisions about which treatment to prescribe.

**Fig. 3:**
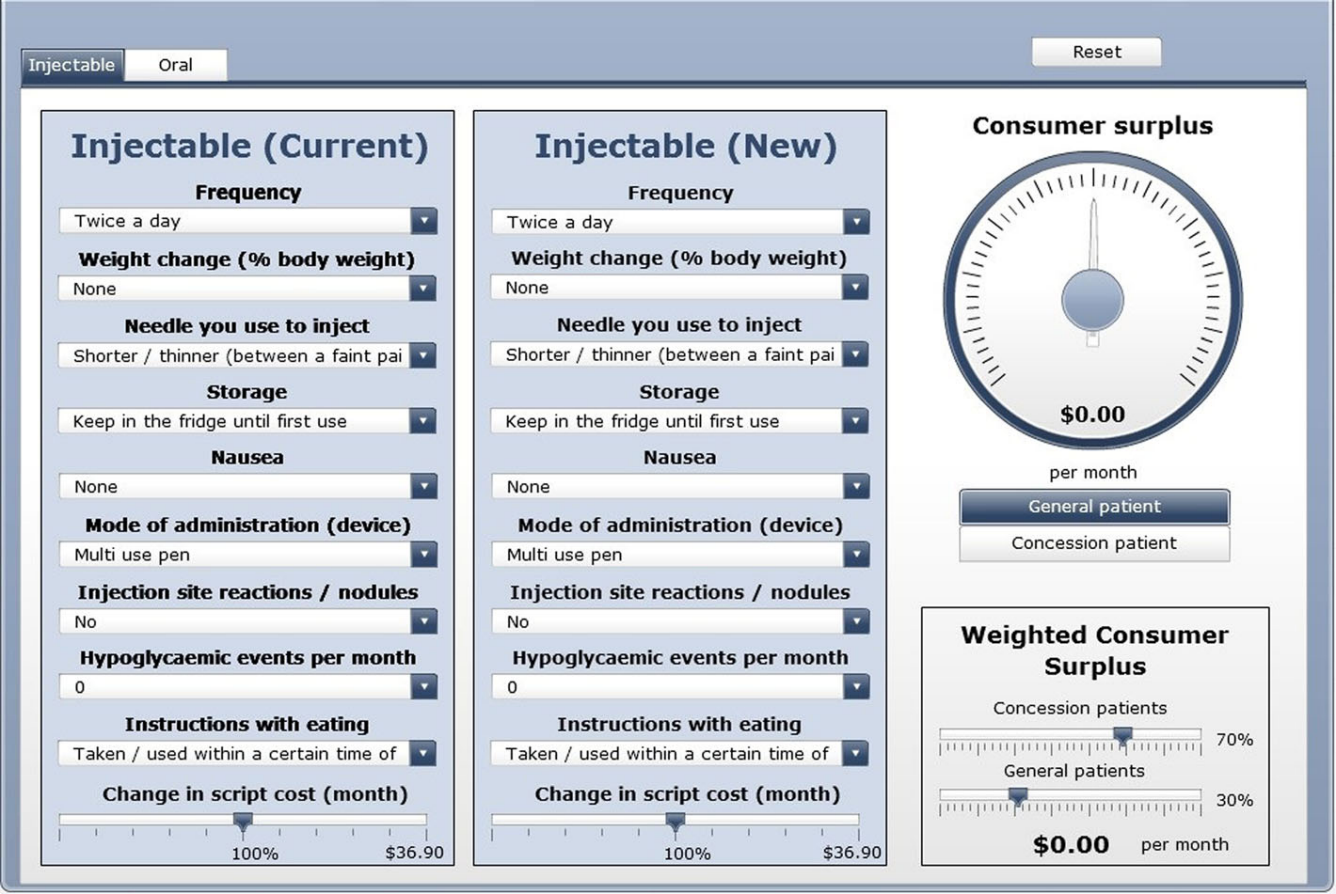
Decision Support System

The DSS was constructed in flash (presented as a pdf file) and provided a simple user interface. A snapshot of the Decision Support System is shown in Fig. 3.

## Discussion

The use of discrete choice experiments in reimbursement submissions is not new in Australia, with Davey et al. 1998 [29] successfully using WTP for insulin lispro, and the Pharmaceutical Benefits Advisory Committee (PBAC) rejecting a WTP claim for Victoza (liraglutide) based on a Scandinavian study by Jendle et al. 2012 [30]. As such, this study investigated the key drivers of choice for T2DM treatments for Australian patients and was designed for presentation to the PBAC as part of the exenatide 2 mg once-weekly (Bydureon) reimbursement dossier.

Bydureon is an extended release microspheres formulation of exenatide, which has been listed on the PBS since 2010 in the twice-daily form (Byetta). Bydureon significantly reduces injection frequency versus Byetta from two per day to one per week, but had not been listed on the Pharmaceutical Benefits Scheme (PBS) despite three reimbursement submissions across 2011 to 2013. This absence on the PBS was in part due to the reimbursement system in Australia, where under the National Health Act, the PBAC cannot grant price premiums for medicines without a significant improvement in efficacy or reduction of toxicity over the alternative therapy.

In the PBAC submission, it was argued that the availability of a once-weekly injectable medicine for T2DM would improve adherence and therefore blood glucose levels and long-term outcomes, particularly in Aboriginal and Torres Strait Islander people who are almost four times more likely than non-Indigenous Australians to have diabetes or pre-diabetes [31]. However, a data gap existed as the participants and circumstances of use in the clinical studies were not sufficiently representative of the proposed population and were unlikely to provide surety to the PBAC that adherence and outcomes would be improved.

Our research demonstrated the importance to Australian patients of a reduced injection frequency, with the consumer surplus considered a surrogate for adherence. The PBAC recommended Bydureon on the basis of potential health benefits from likely improved adherence by a small number of high clinical need populations. Bydureon was subsequently listed on the PBS and greater than fifteen thousand patients now access Bydureon for treatment of their T2DM [32, 33].

This positive recommendation reaffirms the value of local and applicable research in Australian reimbursement submissions, and the increasing importance of providing a voice for the patient during funding decisions.

As with all studies, there are limitations and the results should be interpreted within the context of these limitations. Firstly, as the survey was conducted online, a number of inherent limitations exist. Literacy and language skills were not tested and participants may not have had an adequate understanding of the choice tasks. To adjust for this, a pilot was conducted online with a sample of patients using targeted feedback and adjustments made to ensure logic and understanding. Participants who completed the final survey under a certain time limit, and/or clicked the same response or pattern of responses through the choice tasks, or scored below a certain mark on question regarding understanding of the task were removed from the analysis. Secondly, the diagnosis was not verified by a clinician, and relied on self-reporting by participants. Blinded screening questions (disease and medication based) were incorporated to ensure only patients with a genuine diagnosis meeting the eligibility criteria participated. Nonetheless, the final sample may not accurately represent the diabetes population. Lastly, patients were asked to evaluate certain hypothetical treatment scenarios, which may not necessarily reflect the choices they would make in a real setting.

## Conclusion

This study aimed to investigate the key drivers of choice for type 2 diabetes treatments. A sample of 171 patients (including 58 exenatide patients and 113 oral medicines patients) completed an online survey using state of art choice experiments. In the survey patients were asked to trade off both injectable and oral treatments with different features to ascertain what was most important to them. The most important features of treatment to both exenatide and oral glucose-lowering medicines patients were weight change, frequency and nausea. It is not surprising that weight gain was a key driver of participant choice. Many diabetic patients struggle with their weight and are often actively encouraged to lose weight as part of their disease management. However, often the use of antidiabetic medications are associated with weight gain, which may conflict patients (and impact adherence) and complicate diabetes management.

The results were integrated into a Decision Support System that enables stakeholders to perform scenario analysis. The scenarios presented for the different markets (injectable and oral) measured total willingness to pay (consumer surplus) as a main outcome. The injectable market findings suggest that once-weekly injectable treatment generates an additional benefit over the current treatment twice-daily injectable exenatide, equivalent to a weighted willingness to pay of $22.35 per month. Similar calculations are presented for the oral market. Intuitively, the oral market results show oral treatments are strongly preferred to injectable testaments.

These findings may provide guidance for clinicians making therapeutic decisions regarding T2DM treatments and provide a better understanding of what patients prefer and value in their treatment. This study shows that patients highly value the avoidance of injections, with once-weekly dosing clearly preferred over twice-daily dosing. Of the other attributes, weight loss is preferred, as is the avoidance of nausea. Consideration of patient preference is important when making therapeutic decisions and can improve health outcomes. This type of analysis has a role in health technology assessment and has been presented to PBAC during their assessment of new therapies.
